# HIV and Co-Infections: Updates and Insights

**DOI:** 10.3390/v15051097

**Published:** 2023-04-29

**Authors:** Francesco Di Gennaro, Alessandra Vergori, Davide Fiore Bavaro

**Affiliations:** 1 Department of Precision and Regenerative Medicine and Ionian Area (DiMePRe-J), Unit of Infectious Diseases, University of Bari “A. Moro”, Polyclinic Hospital, 70124 Bari, Italy; davidebavaro@gmail.com; 2 HIV/AIDS Department, National Institute for Infectious Diseases Lazzaro Spallanzani-IRCCS, 00149 Rome, Italy; alessandra.vergori@inmi.it

Co-infections are frequent in HIV patients; some of them may be AIDS-defining, while others share the same underlying virus mechanism. Others may be asymptomatic, causing or influencing HIV illness in a minor way. HIV co-infections with bacterial, viral, or parasitic microorganisms, on the other hand, may affect the natural history of HIV infection and vice versa. This Special Issues of *Viruses* focuses on various aspects of HIV and its co-infections. Eleven papers are included, each focusing on a different issue in an essential field of applied research, furthering the scientific knowledge on this important topic.

Vassallo and colleagues [1], in an extensive study, showed the impact of combination antiretroviral treatment (cART) simplification on immune activation. In their data, collected from up to 2 years of follow-up, they describe how cART simplification to dual therapy was associated with macrophage activation despite successful virological control. This was more pronounced in those at risk of immune activation. Pinetti, on a pembrolizumab compassionate-use basis, describes the experience of the Italian National Institute of Infectious Diseases in the treatment of progressive multifocal leukoencephalopathy (PML) in people living with HIV [2]. PML is a severe demyelinating disease caused by the reactivation of poliomavirus JC (JCV), which mainly occurs in immunosuppressive conditions such as those cause by AIDS. The mechanisms by which JCV reactivates and damages the brain parenchyma are currently poorly understood, and we already know we have few therapeutic options. In their experience, pembrolizumab (PEM), a monoclonal antibody that inhibits the programmed cell death protein 1 (PD-1), is currently approved for the treatment of some cancers. PD-1 expression is upregulated in the CD4+ and CD8+ cells of patients with PML, particularly on the JC virus, and the rationale for its use in the treatment of PML is that the inhibition of PD-1 could potentially be associated with a better anti-JCV-specific response, with consequent JCV clearance and neurological improvement. Unfortunately, two out of five patients died, and more data are needed in order to identify predictors of response to therapy.

COVID-19 severely impacted health systems, especially the most fragile ones, and to investigate the association between radiological changes and poor COVID-19 outcomes among HIV-positive patients from Central and Eastern Europe, Kowalska and colleagues [3] performed a very interesting and well-structured study. They underline the role of ART on COVID-19 outcome; in fact, based on their data, being on antiretroviral therapy decreased the odds of poor COVID-19 outcomes, while having a comorbidity or either typical or atypical radiological changes increased the risk of poor COVID-19 outcomes. In line with these data, Vergori and colleagues reported in a large study how HIV infection did not seem to negatively impact COVID-19-related inflammatory states and immunity [4].

Several studies have dealt in great detail with HCV diseases in PLWHIV, tackling different aspects of the co-infection. In fact, Li and collegues [5], in a large Cinese study, underline the high risk of end-stage liver disease (ESLD) with concomitant rapid progression of liver fibrosis in HIV/HCV co-infected individuals compared with HCV mono-infected patients, with a higher risk of death due to the HIV/HCV co-infection. Moreover, Popping [6] stressed the role of knowing the transmission dynamics of HCV for targeted prevention strategies. Totaro and colleagues [7] underline how migrants in Italy have low HCV knowledge, confirming migrants as a population with a higher risk of infection. These studies can help to develop education programmes, integrated care, and screening among migrants, which could be an effective strategy. Furthermore, very interesting is the experience of Yin [8], who underlines how intestinal mycobiome dysbiosis may play an important role in the advancement of HIV- and HCV-infected patients. In fact, from their data, it was discovered that low-CD4 + T-cell patients in the HIV group and high-ALT-level patients in the HCV group had a more chaotic fungal community with a possible worse health outcome.

Semmani [9], reporting data from Algeria, maps the epidemiology of Cryptosporidium in HIV/AIDS patients, contributing to improved occurrence and molecular characterization. Cryptosporidium species and subtype families are prevalent in Algerian HIV patients.

An interesting paper came from an Italian HIV Referral Hospital, when Malaglino [10] and collegues investigated the potential impact of the presence of anti-hepatitis B (HBV)c antibodies (HBcAb positivity) on the control of HIV viremia in patients living with HIV (PLWH) who switched to two-drug antiretroviral therapy (2DR) containing lamivudine (3TC) (2DR-3TC-based). From their experience, the presence of HBcAb positivity could favour the emergence of HIV viral rebound by nearly 54 percent during the entire study follow-up after switching to 2DR-3TC. Thanks to Nazim and colleagues [11], HIV in children was also treated. In fact, they demonstrated how the presence of EBV/CMV co-infection can affect HIV viral loads and the expression of certain cytokines (IFN-γ and TGF-β1), which may affect the disease dynamics of HIV in infected children.

I am very proud of this Special Issue and believe that it is a source of significant value and inspiration for the clinical and academic communities around the world.
